# Immunotherapy Resistance in Glioblastoma

**DOI:** 10.3389/fgene.2021.750675

**Published:** 2021-12-17

**Authors:** Elaina J. Wang, Jia-Shu Chen, Saket Jain, Ramin A. Morshed, Alexander F. Haddad, Sabraj Gill, Angad S. Beniwal, Manish K. Aghi

**Affiliations:** ^1^ Department of Neurological Surgery, The Warren Alpert School of Medicine, Brown University, Providence, RI, United States; ^2^ Department of Neurological Surgery, University of California, San Francisco, San Francisco, CA, United States

**Keywords:** glioblastoma, immunotherapy, resistance, immunoprivilege, checkpoint inhibitors, vaccine, CAR (chimeric antigen receptor) T cells, virotherapy

## Abstract

Glioblastoma is the most common malignant primary brain tumor in adults. Despite treatment consisting of surgical resection followed by radiotherapy and adjuvant chemotherapy, survival remains poor at a rate of 26.5% at 2 years. Recent successes in using immunotherapies to treat a number of solid and hematologic cancers have led to a growing interest in harnessing the immune system to target glioblastoma. Several studies have examined the efficacy of various immunotherapies, including checkpoint inhibitors, vaccines, adoptive transfer of lymphocytes, and oncolytic virotherapy in both pre-clinical and clinical settings. However, these therapies have yielded mixed results at best when applied to glioblastoma. While the initial failures of immunotherapy were thought to reflect the immunoprivileged environment of the brain, more recent studies have revealed immune escape mechanisms created by the tumor itself and adaptive resistance acquired in response to therapy. Several of these resistance mechanisms hijack key signaling pathways within the immune system to create a protumoral microenvironment. In this review, we discuss immunotherapies that have been trialed in glioblastoma, mechanisms of tumor resistance, and strategies to sensitize these tumors to immunotherapies. Insights gained from the studies summarized here may help pave the way for novel therapies to overcome barriers that have thus far limited the success of immunotherapy in glioblastoma.

## Introduction

Glioblastoma (GBM) is the most common cause of primary brain malignancy, accounting for 27% of all brain tumors and 80% of malignant brain tumors (Ostrom et al., 2015). The current standard of care (SOC) for primary GBM is maximally safe surgical resection followed by concurrent radiotherapy and temozolomide (TMZ) for 6 weeks and then adjuvant TMZ for 6 months (Fernandes et al., 2017). However, despite treatment, median survival remains low with a 2-year survival rate of under 30% (Stupp et al., 2005) and recurrence occurring in over 90% of high-grade glioma patients (Choucair et al., 1986).

The recent use of immunotherapies, such as immune checkpoint inhibitors and autologous T cells expressing chimeric antigen receptors (CAR), to successfully treat various solid and hematologic malignancies has led to growing interest in applying similar methods to GBM. Nivolumab, an anti-programmed death-1 (PD-1) antibody, and ipilimumab, an anti-cytotoxic T-lymphocyte associated-protein 4 (CTLA-4) antibody, have led to improvements in survival when used in stage III and IV melanoma patients (Wolchok et al., 2017). Autologous T cells genetically engineered to express CAR specific for CD19 on B cells have been used to treat hematologic malignancies (Kalos et al., 2011). Despite successes with other cancers, similar checkpoint inhibitor and T cell therapies applied to GBM have not seen the same level of success.

This review aims to delineate the multiple avenues of immunotherapy that have been tested in glioblastoma treatment, including checkpoint inhibitors, vaccines, adoptive transfer of effector lymphocytes, and oncolytic virotherapy to stimulate an anti-tumoral immune response. We will also address the mechanisms of primary and secondary resistance seen in the immunologically unique environment of the CNS. Finally, we will discuss strategies to overcome the immunosuppressive tumor microenvironment and subsequently sensitize tumors to the immune response.

### Immunotherapies

#### Immune Checkpoint Inhibitors

Immune checkpoints are naturally occurring co-inhibitory receptors expressed on the surface of T cells that play an important role in down-modulating the immune response and promoting self-tolerance (Pardoll, 2012). While these receptors initially evolved to prevent the development of autoimmunity and maintain immune homeostasis, they have been found to be upregulated in various forms of cancer promoting immune tolerance to tumor cells (Pardoll, 2012). To this end, inhibitors targeting checkpoint molecules such as CTLA4 and PD1 have been used to successfully treat patients with solid tumors. In the following section, we will be covering well-known checkpoint inhibitors and their successes and pitfalls in treating various cancers (Table 1).

**TABLE 1:** Past and present phase II/III clinical trials with ICIs in glioblastoma.

Clinical trial	Duration	Phase	Target	Treatment	Control	Indication	Outcome	References
ISRCTN84434175 Ipi-Glio	2018-	II	CTLA4	Ipilimumab + TMZ (*n* = 80)	TMZ (*n* = 40)	ndGBM	Ongoing	(Brown et al., 2020)
NCT02017717 CheckMate 143	2014-	III	PD-1	Nivolumab (*n* = 184)	Bevacizumab (*n* = 185)	rGBM	OS-12 months: 42%	(Reardon et al., 2020)
NCT02550249	2015–2017	II	PD-1	Neo- and adjuvant nivolumab (*n* = 30)	None	ndGBM, rGBM	OS: 7.3 months	(Schalper et al., 2019)
NCT02617589 CheckMate 498	2016–2021	III	PD-1	Nivolumab + RT (*n* = 280)	TMZ + RT (*n* = 280)	ndGBM	Non-improved OS	(Sampson et al., 2016)
NCT02667587 CheckMate 548	2016-	III	PD-1	Nivolumab + RT + TMZ	Placebo + TMZ + RT	ndGBM	Ongoing	BMS press release
NCT02337491	2015–2020	II	PD-1	Pembrolizumab + bevacizumab (*n* = 50)	Pembrolizumab (*n* = 30)	rGBM	PFS-6months 26 vs 6.7%	(Nayak et al., 2021)
NCT02337686	2015–2020	II	PD-1	Pembrolizumab + Surgery (*n* = 15)	None	rGBM	PFS-6: 53%	(De Groot et al., 2018)
NCT03174197	2017-	II	PDL1	Atezolizumab + TMZ (*n* = 50)	None	ndGBM	OS: 17.1 mo	(Weathers et al., 2020)
NCT03291314 GLIAVAX	2017-	II	PDL1	Avelumab + axitinib (*n* = 54)	None	rGBM	PFS-6 months: 18%	(Neyns et al., 2019)
NCT02336165	2015	II	PDL1	Durvalumab + RT (*n* = 40)	None	ndGBM	OS-12 months: 60%	(Reardon et al., 2019)
NCT03047473	2017–2021	II	PDL1	Avelumab (*n* = 30)	None	ndGBM	Ongoing	(Jacques et al., 2018)

##### Anti-CTLA4

The first checkpoint inhibitor approved for clinical use in cancer patients targeted CTLA4 (Hodi et al., 2003; Phan et al., 2003; Hodi et al., 2010; Hodi et al., 2003; Phan et al., 2003; Hodi et al., 2010), an inhibitory receptor expressed on regulatory T cells (Tregs), CD4, and CD8 T cells (Chan et al., 2014). Blockade of CTLA4 has been shown to increase the infiltrative T cell and decrease Treg response to tumor cells (Curran et al., 2010) by allowing the co-stimulatory receptor CD28 to bind CD80 (B7.1) and CD86 (B7.2) expressed on antigen-presenting cells (APC) (Linsley et al., 1994). Under homeostatic conditions, the interaction between CD28 and B7 provides a crucial second signal to activate T cells (Lenschow et al., 1996). This mechanism is well demonstrated by the rampant autoimmunity seen in CTLA4 knockout murine models (Tivol et al., 1995; Waterhouse et al., 1995).

Blockade of CTLA4 using the checkpoint inhibitor ipilimumab has been shown to improve survival in patients with unresectable stage III or IV melanoma when compared to use of a peptide vaccine alone (Hodi et al., 2010), and similar anti-CTLA4 therapies are currently being studied in clinical trials for cervical cancer, bladder cancer, and soft tissue sarcoma. In non-small cell lung cancer (NSCLC), the combination of anti-CTLA4 and anti-PD1 has been shown to delay time to deterioration compared to chemotherapy and resulted in an overall response rate of 30% in the CheckMate 568 study of 288 patients with stage IIIB/IV NSCLC (Ready et al., 2019; Reck et al., 2021). In preclinical murine models of GBM, CTLA4 blockade has been shown to decrease Treg populations and improve long-term survival (Fecci et al., 2007; Grauer et al., 2007). In patients with melanoma metastases to the brain, ipilimumab reaches a response rate of 18% in neurologically asymptomatic patients not on steroids and 5% in symptomatic patients taking steroids (Margolin et al., 2012). The use of ipilimumab with an anti-PD1 inhibitor in metastatic melanoma to the brain has also been shown to have improved intracranial efficacy than either monotherapy, likely via significant increase of CD8^+^ T cell migration to the brain (Taggart et al., 2018).

In GBM, the combination of ipilimumab and bevacizumab, a monoclonal antibody that inhibits vascular endothelial growth factor (VEGF), has been tested in 20 patients and demonstrated a 31% partial response rate with adverse events in 2 patients (Carter et al., 2016). While this suggests an overall benign safety profile for the combination of these two drugs to treat GBM, it has been noted in the literature that the mixture of newly diagnosed GBM (ndGBM) and recurrent GBM (rGBM) patients in the study, the use of radiographic response as a determinant of response rather than clinical status, and the higher rates of Grade 3 toxicity in combination therapy compared to bevacizumab alone (35 versus 11%) warrant closer investigation (Tini and Pirtoli, 2016). This has led to a formal phase I trial testing the maximally safe dose in three separate conditions: ipilimumab and TMZ, nivolumab and TMZ, and the combination of all three drugs in patients with gliosarcoma or ndGBM (National Cancer Institute (NCI), 2020).

Ipilimumab is also being tested as neoadjuvant treatment in combination with anti-PD1 inhibitor nivolumab for patients with surgically resectable GBM (MD PYW, 2021), and the same combination has been shown to be safe via intratumoural and intracavitary administration in a phase I trial for rGBM patients (Schwarze et al., 2020). In the ongoing Ipi-Glio trial, ipilimumab is being tested in combination with TMZ versus TMZ alone in rGBM patients with results pending (Brown et al., 2020).

##### Anti-PD1/PDL1

The successes in targeting CTLA4 led to the development of similar antibodies against the checkpoint molecule PD1 and its ligand PDL1. PD1 is predominantly expressed on activated B and T cells, and it counters CD28-mediated stimulatory processes by binding its ligands PDL1 and PDL2 (Latchman et al., 2001; Keir et al., 2008). Much like the downstream consequences of CTLA and B7 binding, the interaction between PD1 and its ligands subsequently inhibits T cell activation and proliferation (Sharpe and Pauken, 2018), a mechanism highlighted by the presence of lymphoproliferation and spontaneous multi-organ autoimmunity in PD1 deficient murine models (Nishimura et al., 1998; Wang et al., 2005). PDL1 is not only expressed on immune cells but also on various tissues such as endothelial and epithelial cells, as well as classically ‘immune privileged’ sites such as the eye (Boussiotis, 2016). Its ubiquitous expression and upregulation on tumor cells suggest that PDL1 may play a role in immune evasion (Blank et al., 2005), making the PD1/PDL1 pathway an ideal target for checkpoint inhibition.

Two anti-PD1 inhibitors have been approved for clinical use: nivolumab and pembrolizumab. Initially tested in patients with melanoma, nivolumab was found to increase overall survival (72.9%) at 1 year in patients with metastatic melanoma compared to patients receiving dacarbazine (42.1%) (Robert et al., 2015). The combined use of nivolumab and ipilimumab (58%) was subsequently shown to increase rate of overall survival at 3 years compared to ipilimumab alone (34%) in a phase III trial from 2017 conducted in patients with advanced melanoma.^345^ However, similar findings have not been reproduced for nivolumab use in glioblastoma.

There have been three phase III trials testing the use of nivolumab in GBM: Checkmate 143, Checkmate 498, and Checkmate 548. Checkmate 143 tested use of nivolumab versus bevacizumab in patients with rGBM and found comparable overall survival (42%) in the two groups, with a higher objective response rate to bevacizumab (23.1%) than to nivolumab (7.8%) (Reardon et al., 2020). RNA sequencing of human glioma tissue after neoadjuvant nivolumab treatment did demonstrate increased immune cell infiltrate, T cell receptor clonal diversity, and expression of chemoattractant transcripts such as *CCL4* and *CCL3L1* compared to pre-treatment tissue (Schalper et al., 2019). However, despite the promotion of immune surveillance, nivolumab use did not significantly affect patient outcome.^3738^ Checkmate 498 tested nivolumab and radiation versus SOC TMZ and radiation in treatment-naïve, MGMT-unmethylated patients and found non-improved overall survival in the nivolumab-treated cohort (Sampson et al., 2016). Checkmate 548 tested the use of nivolumab and SOC to placebo and SOC in ndGBM patients with MGMT methylation but was unable to meet its primary endpoint of overall survival (Bristol-Myers Squibb, 2020a).

Pembrolizumab use has had a similar trajectory to that of nivolumab, finding limited success in GBM compared to melanoma. In a phase III trial testing pembrolizumab to ipilimumab for advanced melanoma patients (KEYNOTE-006), pembrolizumab use was associated with increased overall survival (Schachter et al., 2017). As adjuvant therapy in patients with resected stage III melanoma, pembrolizumab use (75.4%) continued to be associated with longer recurrence-free survival at 1 year than placebo (61%) (Eggermont et al., 2018). However, these findings did not translate to GBM, and a phase II trial using pembrolizumab alone versus with bevacizumab in patients with rGBM did not find a significant therapeutic benefit in either group (Nayak et al., 2021).

Atezolizumab, avelumab, and durvalumab are three anti-PDL1 inhibitors that have been approved for clinical use. Atezolizumab has seen promising results in both GBM and other cancers. In the IMvigor130 trial in urothelial cancer patients, the combination of atezolizumab and platinum-based chemotherapy was found to prolong progression-free survival compared to placebo and chemotherapy (Galsky et al., 2020). Atezolizumab was then found to be well-tolerated in rGBM patients, particularly in patients with a high peripheral CD4^+^ T cell count, (Lukas et al., 2018), and a phase II trial found the combination of atezolizumab TMZ, and radiation in ndGBM patients to be tolerable and efficacious with a median OS of 19 months and median PFS of 10.6 months (M.D. Anderson Cancer Center, 2021). However, avelumab has not seen a similar level of success in GBM, having failed to meet the threshold for justifying further investigation in the GLIAVAX trial testing axitinib and avelumab combination use in rGBM patients following SOC (Neyns et al., 2019). Durvalumab has been FDA approved for bladder cancer and non-small cell lung cancer (NSCLC) and is currently being tested in a phase II trial for newly diagnosed unmethylated GBM patients (Ludwig Institute for Cancer Research, 2021).

##### Anti-LAG3 (CD223)

Lymphocyte activation gene-3 (LAG3) is a cell surface molecule that associates with the CD3/T cell receptor (TCR) complex to competitively bind MHC II molecules on antigen-presenting cells (APC), subsequently inhibiting immune cell proliferation (Long et al., 2018). Constant antigen exposure in the tumor microenvironment has been shown to upregulate LAG3 expression and contribute to immune cell exhaustion (Andrews et al., 2017). Its pervasive and aberrant expression in the tumor microenvironment has made it a target of interest in cancers that have seen limited success with established ICIs.

Preclinical data in murine GBM models has revealed improved survival in LAG3 knockout mice receiving anti-PD1 treatment compared to wild-type (WT) mice treated with anti-PD1, suggesting that LAG3 inhibition may potentiate the anti-tumoral effect of anti-PD1 (Harris-Bookman et al., 2018). The use of relatlimab, an anti-LAG3 monoclonal antibody, and nivolumab in combination is currently being tested in several clinical trials for GBM, hematologic malignancies, and other advanced solid tumors (Puhr and Ilhan-Mutlu, 2019). Of the anti-LAG3 drugs under development, relatlimab is the only to undergo phase III trials, currently for metastatic melanoma (Bristol-Myers Squibb, 2020b).

#### Vaccines (Table 2)

##### Peptide Vaccines

Peptide vaccines utilize in vitro-synthesized peptides to induce a lasting anti-tumor immune response (Kumai et al., 2017). The targets of peptide vaccines are either tumor-associated antigens (TAA) or tumor-specific antigens (TSA) (Calvo Tardón et al., 2019). TAAs are expressed in both non-malignant and malignant tissue but have higher expression in malignant tissue, while TSA are exclusively expressed in malignant tissue (Calvo Tardón et al., 2019). While TSAs are most often derived from non-synonymous single nucleotide variants (SNV), which are patient-specific, there has also been a push to use TSAs derived from alternative sources such as frameshift mutations, splice variants, fusion proteins, and endogenous retroelements, which have the benefit of being more likely to be shared among tumors and less likely to be patient-specific (Smith et al., 2019).

**TABLE 2:** Past and present phase II/III clinical trials with vaccines in glioblastoma.

Clinical trial	Duration	Phase	Target/Lysate	Treatment	Control	Indication	Outcome	References
NCT01498328 ReACT	2011–2016	II	EGFRvIII	Bevacizumab + Rindopepimut (*n* = 33)	Bevacizumab + KLH (*n* = 35)	rGBM	PFS-6months: 27 vs 11%	(Reardon et al., 2015)
NCT00458601 ACT III	2007–2016	II	EGFRvIII	SOC + Rindopepimut + GM-CSF (*n* = 65)	None	ndGBM	PFS-5.5 months: 66%	(Schuster et al., 2015)
NCT01480479 ACT IV	2011–2016	III	EGFRvIII	Rindopepimut + GM-CSF + TMZ (*n* = 371)	KLH + TMZ (*n* = 374)	ndGBM	OS: 20.1 vs 20 months	(Weller et al., 2017)
NCT00643097 ACTIVATe	2007–2016	II	EGFRvIII	PEP-3-KLH conjugate + GM-CSF (*n* = 18)	TMZ (*n* = 17)	ndGBM	PFS-6 months: 67%	(Sampson et al., 2010)
NCT01920191	2013–2016	II	TAA	SOC + IMA950/poly-ICLC (*n* = 19)	None	ndGBM	OS: 19 months	(Migliorini et al., 2019)
NCT00766753	2006–2016	II	TAA	αDC1 + poly-ICLC (*n* = 22)	None	rGBM	PFS-12 months: 40.9%	(Okada et al., 2011)
NCT02078648	2014–2018	II	TAA	SL-701 + poly-ICLC + bevacizumab (*n* = 74)	None	rGBM	OS-12: 37%	(Peereboom et al., 2018)
NCT00293423	2013–2017	II	Autologous peptides	HSPPC-96 (*n* = 41)	None	rGBM	OS-6 months: 90.2%	(Bloch et al., 2014)
NCT00905060	2009–2014	II	Autologous peptides	HSPPC-96 + TMZ (*n* = 46)	None	ndGBM	OS: 23.8 months	(Bloch et al., 2017a)
NCT01814813	2013–2017	II	Autologous peptides	HSPPC0-96 + bevacizumab (*n* = 59)	Bevacizumab (*n* = 31)	rGBM	OS: 7.5 vs 10.7 months	(Bloch et al., 2017b)
NCT00045968	2006–2016	III	Tumor lysate	DCVax-L + TMZ (*n* = 232)	Autologous PBMC + TMZ (*n* = 99)	ndGBM	PFS-2 months: 46.2% PFS-3 months: 25.4%	(Liau et al., 2018)

Vaccines targeting TAAs have demonstrated non-improved overall survival in phase III trials for pancreatic cancer (Middleton et al., 2014) and renal cell carcinoma (Rini et al., 2016), but the use of a peptide vaccine against three TAAs (EphA2, IL13Rα2, and survivin) in children with recurrent high-grade gliomas was well tolerated and demonstrated a median PFS at 4.1 months and median OS at 12.9 months (Pollack et al., 2016). IMA950, a multi-peptide vaccine containing 11 tumor-associated peptides (Rampling et al., 2016), was well-tolerated in a phase I trial of ndGBM patients (Rampling et al., 2016) and shown to induce immunogenicity in the form of CD8^+^ T cell responses in 63.2% of ndGBM and grade III astrocytoma patients (Migliorini et al., 2019). However, unlike the synergy demonstrated between anti-LAG3 and anti-PD1 inhibitors, IMA950 vaccination did not improve response to bevacizumab in high grade glioma patients, and there were no significant differences in median PFS or OS between vaccinated and control patients (Boydell et al., 2019).

Epidermal growth factor receptor variant III deletion mutation (EGFRvIII) is the most well-studied TSA in GBM, and there have been several clinical trials conducted focusing on rindopepimut, a peptide vaccine targeting EGFRvIII (Swartz et al., 2014). The ReACT and ACT III are two phase II trials that have tested rindopepimut efficacy in targeting GBM. In the ReACT trial, combined treatment with rindopepimut and bevacizumab was tested against placebo in rGBM patients and found to have increased PFS at 6 months (27 vs 11%) (Reardon et al., 2015). The ACT III trial found promising PFS of 66% at 5.5 months and OS of 26% at 36 months in patients treated with rindopepimut and adjuvant TMZ after resection of EGFRvIII^+^ GBM (Schuster et al., 2015). However, the follow-up ACT IV phase III trial testing rindopepimut versus control did not find increased overall survival in ndGBM patients (Weller et al., 2017).

The isocitrate dehydrogenase 1 (IDH1) mutation is another target of interest due to its expression in over two-thirds of all low-grade gliomas (Sun et al., 2013). Vaccines designed with peptides containing the R132H mutation have elicited mutation-specific CD4^+^ responses in mice expressing human MHC class I and II (Schumacher et al., 2014). A similar peptide vaccine against R132H was safely tested in a phase I trial of grade III and IV astrocytoma patients and demonstrated 82% PFS at 2 years in patients with immune responses (Platten et al., 2021).

Heat shock proteins (HSP) have also been utilized to stimulate anti-tumor immune responses due to their intrinsic ability as molecular chaperones to carry peptides that are subsequently cross-presented to the immune system (Shevtsov and Multhoff, 2016). Heat shock protein-peptide complexes (HSPPC) have been developed using tumor-derived HSP to successfully generate CD4^+^ and CD8^+^ responses in mouse models of mammary and lung tumors (Manjili et al., 2003) (p110), (Wang et al., 2003), In GBM, HSPPC-96 is one such vaccine designed with gp96, a multifunctional HSP capable of inducing both innate and tumor-specific adaptive immunity (Schild and Rammensee, 2000). A phase I trial in ndGBM patients demonstrated a higher median OS for patients with high tumor-specific immune responses (>40.5 months) after receiving HSPPC-96 compared to patients with low responses (14.6 months), as well as a PFS at 6 months of 89.5% (Ji et al., 2018). Patients with rGBM who received HSPPC-96 in a phase II trial were found to have a median OS of 42.6 weeks, with 90.2 and 29.3% of patients alive at 6 and 12 months respectively. (Bloch et al., 2014).

##### Dendritic Cell Vaccines

DC vaccines utilize the cell’s potent antigen presentation capabilities to elicit anti-tumor immune responses (Filley and Dey, 2017). Autologous DCs are harvested from the patient and stimulated with tumor antigens *ex vivo* before re-infusion (Tacken et al., 2007). DCs can be pulsed with one or multiple antigens – both approaches have been explored in clinical trials.

Two phase I trials that pulsed DCs with Wilms’ tumor 1 (WT1) and six GBM TAAs respectively have found these vaccines to be safe in rGBM and ndGBM patients (Phuphanich et al., 2013; Sakai et al., 2015). In the latter study utilizing multiple antigens, six patients remained tumor-free at 40 months follow-up (Phuphanich et al., 2013). A phase II trial testing α-type 1 polarized DCs also demonstrated a sustained response in one rGBM patient and PFS at 12 months in nine out of 22 patients (Okada et al., 2011). The use of cytomegalovirus phosphoprotein 65 RNA (CMV pp65) to prime DCs is also an active area of interest given the expression of pp65 in human glioma samples (Cobbs et al., 2002). DCs pulsed with cytomegalovirus phosphoprotein 65 RNA (CMV pp65) have been tested in ndGBM patients, achieving a median PFS and OS of 25.3 and 41.1 months respectively with four out of eleven patients staying progression-free at 59 months (Batich et al., 2017).

DCVax-L is another DC-based vaccine that pulses dendritic cells with autologous tumor lysate. A phase I and II trial testing autologous DC-tumor vaccine therapy in both recurrent and newly diagnosed GBM patients achieved a median survival of 525 days with lymphopenia and reversible elevations in AST/ALT as the only two reported adverse effects (Chang et al., 2011). As a result, a phase III trial was conducted that combined DCVax-L with SOC for 331 ndGBM patients, which revealed a median overall survival of 23.1 months from surgery and a 3-years survival of 46.4% (Liau et al., 2018).

#### Adoptive T Cell Therapy (Table 3)

The adoptive transfer of tumor infiltrative lymphocytes (TIL) has been extensively studied in GBM. Resected tumor specimen and lymphocytes are taken out of the patient and co-cultured *in vitro*, and lymphocytes reactive against TAAs are selected for as TILs and subsequently expanded prior to infusion back into the patient (Wang et al., 2020). The safety of this method was established in a study of six recurrent glioma patients who received autologous TILs that were expanded *in vitro* with recombinant IL-2. In this study, the only complications were low grade fevers, asymptomatic hydrocephalus, and asymptomatic cerebral swelling, and half of the patients had a partial response at 6 months while 1 patient had complete response at 45 months (Quattrocchi et al., 1999). Although TILs are not typically genetically modified at baseline in adoptive transfer, there is also an ongoing phase I trial evaluating the safety of using TILs transduced to express PD1 antibody in glioma patients (MD YY, 2021). Other than TILs, the adoptive transfer of lymphokine-activated killer (LAK) cells, which are peripheral mononuclear blood cells (PBMC) incubated with IL-2 *in vitro*, has revealed higher median survival rates for rGBM patients compared to those that undergo reoperation for recurrence (Dillman et al., 19972004). The use of LAKs to treat GBM is also currently being studied in a phase II trial (Hoag Memorial Hospital Presbyterian, 2013).

**TABLE 3:** Past and present phase I/II clinical trials with adoptive T cell transfer in glioblastoma.

Clinical trial	Duration	Phase	T Cell	Control	Indication	Objective response	References
NCT00331526	1999–2008	II	Autologous LAK (*n* = 33)	None	ndGBM	NR	(Dillman et al., 2009)
NCT01109095	2010–2018	I	HER2-CAR CMV-T cells (*n* = 16)	None	rGBM	8/16 (50%)	(Ahmed et al., 2017)
NCT02209376	2014–2018	I	EGFRvIII-CAR T cells (*n* = 10)	None	rGBM	1/10 (10%)	(O’Rourke et al., 2017)
NCT00730613	2002–2011	I	IL13Rα2-CAR CTL (*n* = 3)	None	rGBM	2/3 (66%)	(Brown et al., 2015)
NCT02208362	2015–2022	I	IL13BB- CAR Autologous T cells (*n* = 1)	None	rGBM	1/1 (100%)	(Brown et al., 2016)

Chimeric antigen receptor (CAR)-modified T cells have emerged in recent years as a promising avenue of individualized immunotherapy. Autologous T cells are taken from the patient and are engineered to express a synthetic chimeric receptor that can recognize target cells independent of antigen processing and MHC restriction (Maus et al., 2014). CAR T-cell therapy targeting CD19 has demonstrated efficacy in treating acute lymphoblastic leukemia (Maude et al., 2014), and it is currently being studied for use in GBM. HER2-specific CAR T cells have demonstrated antitumor activity in preclinical patient-derived xenografts (Ahmed et al., 2010) and shown to be safe in phase I trials using autologous HER2-specific CAR virus-specific T cells in CMV seropositive patients with HER2-positive rGBM (Ahmed et al., 2017). Other cell surface markers such as EGFRvIII and ephrin-A2 (EphA2) have been found to be efficacious targets for CAR T cells in xenograft models of GBM (Chow et al., 2013; Sampson et al., 2014; Johnson et al., 2015). In patients, CAR T cells targeting EGFRvIII in a phase I trial led to marked expansion of tumor-infiltrating T cells but also increased expression of inhibitory regulatory T cells and upregulation of immunosuppressive markers indoleamine 2,3-dioxygenase 1 (IDO1) and PDL1 (O’Rourke et al., 2017). CAR T cells targeting IL13Rα2 have been shown to be well tolerated via intracranial delivery in 3 patients with rGBM (Brown et al., 2015) and led to regression of multifocal rGBM in both brain and spine for 7.5 months in a patient who received both intracavitary and intraventricular infusions of CAR T cells targeting IL13Rα2 (Brown et al., 2016). To overcome the inherent heterogeneity in GBM, there has also been work studying the use of a trivalent CAR T cell that targets IL13Rα2, EphA2, and HER2 that has demonstrated increased and sustained response in patient-derived xenografts (Bielamowicz et al., 2016).

#### Oncolytic Virotherapy (Table 4)

Oncolytic viruses (OV) have demonstrated much promise in eliciting therapeutic responses in several cancers including GBM. The premise of using OVs is twofold in their ability to selectively infect tumor cells and induce tumor cell lysis while also releasing tumor antigens that elicit an anti-tumor immune response. (Foreman et al., 2017).

**TABLE 4:** Past and present phase II/III clinical trials with oncolytic virotherapy in glioblastoma.

Clinical trial	Duration	Phase	Virus type	Treatment	Control	Indication	Outcome	References
NCT04482933	2021-	II	Herpes simplex virus	HSV G207	None	Recurrent high grade glioma	NR	(MD GKF, 2021)
NCT02798406 CAPTIVE/KEYNOTE 192	2016–2021	II	Adenovirus	DNX 2401 + pembrolizumab (*n* = 49)	None	rGBM	Median OS: 12.5 months	BioSpace Press release
NCT02986178	2017-	II	Poliovirus	PVS-RIPO (*n* = 122)	None	rGBM	NR	(UHSCC, 2021)
NCT04479241 LUMINOS-101	2020-	II	Poliovirus	PVS-RIPO + pembrolizumab (*n* = 30)	None	rGBM	NR	(Sloan et al., 2021)
NCT01174537	NA	I/II	Newcastle disease virus	NDV-HUJ (*n* = 14)	None	rGBM	PFS range: 2–37 weeks OS range: 3–66 weeks	(Freeman et al., 2006)
NA	Published 1998	I/II	Replicating Retrovirus	HSV-tk (*n* = 12)	None	rGBM	OS-12: 25%	(Klatzmann et al., 1998)
NA	Published 1999	I/II	Replicating Retrovirus	HSV-tk (*n* = 48)	None	rGBM	OS-12: 27%	(Shand et al., 1999)
NA	Published 2004	III	Replicating Retrovirus	HSV-tk + SOC (*n* = 124)	SOC (*n* = 124)	ndGBM	OS-12: 50 vs 55% (treatment vs control)	(Rainov, 2000)
NCT02414165	2015–2019	II	Replicating Retrovirus	Toca 511 (*n* = 201)	SOC (*n* = 202)	ndGBM & rGBM	Median OS: 11.1 vs 12.2 mth (treatment vs control)	(Cloughesy et al., 2020)

### Herpes Simplex Virus

The first OV therapy approved by the FDA in the United States was talimogene laherparepvec (tvec), an oncolytic herpesvirus, for advanced melanoma in 2015 (Conry et al., 2018), paving the way for the use of a host of mutated herpesviruses to treat GBM. Many of these mutated constructs are designed to allow preferential infection and lysis of tumor cells, allowing for viral propagation and stimulation of an immune response via release of tumor antigens (Yin et al., 2017). Two main HSV mutants tested in clinical trials for GBM are G207, capable of replicating in only dividing cells, and HSV1716, which can replicate in both dividing and nondividing cells (Immidisetti et al., 2021). There have been three phase I trials that demonstrated the safety and tolerability of G207 in rGBM and ndGBM patients (Markert et al., 2000; Markert et al., 2009; Markert et al., 2014), and similarly, three phase I trials have shown HSV1716 to be tolerable in ndGBM and rGBM patients (Rampling et al., 2000; Papanastassiou et al., 2002; Harrow et al., 2004). Additionally, there was a phase I trial testing intratumoral and peritumoral injection of HSV1716 after resection in pediatric patients with recurrent gliomas that was terminated due to low recruitment (Pediatric Brain Tumor Consortium, 2016). HSV1716 is currently undergoing testing in a phase II trial for children with recurrent high grade glioma (MD GKF, 2021). An alternate version of G207 named G47Δ is currently undergoing phase I and II studies in rGBM patients in Japan (JPRN-UMIN000002661). There is also a phase I trial currently underway testing M032, a genetically engineered HSV expressing IL12 transgene, in rGBM patients (MD JM, 2021).

### Adenovirus

Genetically manipulated adenovirus has also become a popular form of viral therapy in treating GBM. ONYX-015 is an adenovirus construct attenuated via deletion at the E1b locus that was one of the first such constructs to be tested in humans (Kirn, 2001). It was investigated in a 2004 phase I study of 24 rGBM patients who received intratumoral injections with a median survival of 6.2 months (Chiocca et al., 2004). However, attention ultimately shifted to other forms of adenovirus constructs for multiple reasons, including a lack of response to ONYX-015 as a single agent for solid tumors in multiple trials (Kirn, 2001).

There is now much work surrounding DNX-2401, a replication-competent adenovirus that is unable to bind healthy cells with intact retinoblastoma pathways and thus selectively binds tumor cells (Philbrick and Adamson, 2019) (p2401). A phase I trial for DNX-2401 tested dose escalation protocols via either single intratumoral injection or permanently implanted catheter followed by tumor resection for both groups and resulted in 5 (20%) patients with over 3 years of survival in the first group and 2 (17%) patients with over 2 years of survival in the second group (Lang et al., 2018). DNX-2401 has been demonstrated to promote a shift towards the M1 macrophage phenotype in the CSF of treated GBM patients compared with controls, suggesting a treatment-mediated protumoral shift in the immune landscape (Van den Bossche et al., 2018). A phase Ib trial has been completed testing the use of DNX-2401 alone versus in combination with interferon gamma (IFN-γ); while IFN-γ did not provide additional survival benefit, DNX-2401 alone appeared to provide an OS-12 of 33% across 27 patients (Lang et al., 2017). The recently completed phase II CAPTIVE/KEYNOTE 192 trial testing DNX-2401 in combination with pembrolizumab in rGBM patients has reported positive results with a median OS of 12.5 months (DNAtrix, 2021).

### Poliovirus

PVS-RIPO is a replication-competent polio-rhinovirus chimera that selectively infects cells expressing CD155 and demonstrates attenuated neurovirulence via substitution of the native internal ribosome entry site (IRES) for that of rhinovirus (Gromeier and Nair, 2018). Preclinical studies have demonstrated that PVS-RIPO infection may induce dendritic cell and neutrophilic recruitment to the tumor site *in vivo* (Gromeier and Nair, 2018; Mosaheb et al., 2020). A phase I trial testing intratumoral injection of PVS-RIPO in 61 rGBM patients demonstrated a higher OS rate of 21% at 36 months compared to historical controls, with 2 patients surviving for more than 70 months (Desjardins et al., 2018). The favorable results from phase I have led to two ongoing phase II trials testing PVS-RIPO alone in 122 rGBM patients (UHSCC, 2021) and testing PVS-RIPO in combination with pembrolizumab in 30 rGBM patients in the LUMINOS-101 trial. (Istari Oncology, 2021).

### Newcastle Disease Virus

NDV, like much of the viruses discussed here, is capable of selectively infecting and inducing lysis in tumor cells from a variety of cancers (Matveeva et al., 2015). There have been three strains developed and tested in clinical trials over the years: MTH-68, NDV-HUJ, and Ulster. The first reported use of the MTH-68 strain to treat CNS tumors occurred in 1999 in pediatric patients with rGBM. This was followed up with a 2004 study that tested MTH-68 in four patients with high grade glioma that resulted in survival rates ranging from five to 9 years (Csatary and Bakács, 1999; Csatary et al., 2004). NDV-HUJ was tested in a 2006 phase I/II trial of 14 rGBM patients that had been refractory to treatment, with minimal Grade I and II toxicities only and three long-term survivors who all eventually progressed either clinically or radiologically (Freeman et al., 2006). In 2001, Schneider et al. tested autologous tumor cells infected with Ulster NDV and subsequently irradiated in 11 patients after surgical resection and found comparable survival compared to patients who received chemotherapy instead of virotherapy (Schneider et al., 2001). A similar approach was taken in 23 ndGBM patients who underwent maximal resection and received ATV-NDV, an anti-tumor vaccine infected with the Ulster NDV strain; the results demonstrated significantly increased rates of OS-1 (91 versus 45%) and OS-2 (39 versus 11%) compared to the control group (Steiner et al., 2004).

### Reovirus

Reovirus is a double-stranded RNA virus that preferentially infects and lyses tumor cells in part via overactivated Ras signaling pathways that enhance proteolytic viral disassembly in malignant cells (Coffey et al., 1998; Norman et al., 2004; Alain et al., 2007). The application of reovirus for treating tumors was first accomplished via phase I studies on prostate cancer and cutaneous metastases from systemic cancer before eventually being tested on malignant gliomas by Forsyth et al. (2008) Nine of 12 patients were treated for GBM in a dose escalation protocol with no observable adverse events and survival ranging from 6 to 63 weeks (Forsyth et al., 2008). Similar findings were replicated by Kicielinski et al. when applying Reolysin, a wild-type reovirus, to malignant gliomas in a phase I study (Kicielinski et al., 2014). Results were promising, with survival ranging from 14 to 141 weeks, which prompted the designation of orphan drug status to Reolysin for the treatment of malignant glioma by the FDA in 2015 (Jaime-Ramirez et al., 2017). *In vitro* studies have demonstrated that reovirus administration induces DC maturation, stimulates proinflammatory cytokine production, including IFN-alpha, TNF-alpha, and IL-6, and increases NK cell cytolytic activity on tumor cells (Errington et al., 2008). Human studies have confirmed the capacity for reovirus to generate a pro-inflammatory environment, with intravenous delivery of reovirus to brain tumor patients being associated with increased CD8^+^ T cell tumor infiltration, likely attributed to the observed increase in CCL3, CCL4, and ICAM expression, which mediate migration to sites of inflammation (Samson et al., 2018). However, tumors from reovirus-treated patients were also noted to have greater expression of PD-1 and PD-L1 immune checkpoint proteins, highlighting a potential response mechanism by the tumor to counteract the stimulated immune system. While reovirus harbors significant therapeutic utility for the treatment of GBM, further characterization of the tumor’s response to infection is required, as well as consideration for combinatorial treatment with immune checkpoint blockades, such as anti-PD-1/PD-L1. As such, reovirus is no longer being investigated as a monotherapy, and further investigation with other treatment modalities is currently underway (Müller et al., 2020).

### Parvovirus

Parvovirus, specifically H-1 parvovirus (H-1PV), became the focus of many decades of cancer research after discovering that it possesses a natural tropism for human cancer cells in 1961 (Toolan, 1961). The rat is the natural host of H-1PV, and H-1PV has been shown to be nonpathogenic to humans by failing to produce new virus particles and induce cell lysis in normal, non-transformed cells (Angelova et al., 2015). However, it has been shown to infect and cause cell death in a wide range of cancers, including tumors of the bone, brain, breast, colon, lung, pancreas, and skin, as well as hematological disease such as Burkitt lymphoma, cutaneous T-cell lymphoma, and diffuse large B-cell lymphoma (Angelova et al., 2017; Bretscher and Marchini, 2019). The oncotropism of H-1PV involves a myriad of processes, including but not limited to factors essential for viral entry (PKCalpha, CDK1), replication (cyclin A/CDK2, E2F), and maturation (XPO1, PKB, PKC), as well as deficiency of mechanisms necessary to counter viral infection (type I IFN stress response), in tumor cells (Angelova et al., 2015). *In vitro* studies of H-1PV found selective killing of glioma cells via a cathepsin-mediated mechanism, which translated to prolonged survival in glioma-bearing rats treated with intratumoral, intravenous, and intranasal H-1PV inoculation, again via elevated cathepsin activation and activity (Di Piazza et al., 2007; Geletneky et al., 2010). The first clinical trial for GBM was then initiated by Geletneky et al., who found H-1PV (ParvOryx01) to be an immunogenic stimulus in patients with recurrent GBM (Geletneky et al., 2017). Treated patients were found to have strong leukocytic infiltration, predominantly CD8^+^ T cells and, to a lesser degree, CD4^+^ T cells. Additionally, a promising finding was that the increased CD8^+^ T cell population did not coincide with a responsive increase in tumor-invading Tregs that is typical of GBM. This is in line with *in vitro* studies that have found H-1PV to capable of suppressing the activity of Tregs (Moralès et al., 2012). Given these findings, in addition to a case series of patients successfully treated with a combination of NDV, parvovirus, and vaccinia virus, further characterization of the anti-tumor and immune sensitizing effects of H-1PV and investigation of its clinical effects in GBM through a randomized controlled trial is required (Gesundheit et al., 2020).

### Retrovirus

Replicating retroviral vectors have been harnessed for their ability to deliver ‘suicide genes,’ or genes that encode for proteins capable of converting non-toxic into toxic drugs upon delivery of a prodrug (Li et al., 2021). This was the basis of a retroviral vector encoding for HSV thymidine kinase (HSV-tk), which could convert ganciclovir (GCV) into GCV triphosphate, an inhibitor of DNA replication. HSV-tk was tested in 15 GBM patients who received intratumoral injection of HSV-tk followed by intravenous GCV administration, with 1 long-term survivor at 220 weeks (Ram et al., 1997). Twelve rGBM patients exhibited no serious adverse events in a phase I/II study after receiving intratumoral injection of HSV-tk cells intra-operatively, with 25% of patients living longer than 12 months (Klatzmann et al., 1998). A similar study in 13 ndGBM patients demonstrated significantly elevated soluble Fas ligand and IL-12 levels in serum of HSV-tk treated patients versus controls but did not find increased tumor-infiltrating lymphocytes at the resection cavity or activation of T or NK cells (Rainov et al., 2000). Another phase I/II clinical trial in 48 rGBM patients who received intracerebral injection of HSV-tk demonstrated no serious adverse events or evidence of virus in the serum or tissue at time of repeat resection (Shand et al., 1999). However, a phase III trial studying the effects of HSV-tk and GCV treatment in 248 ndGBM patients found no significant difference in PFS and median survival between treated and control patients, which was hypothesized to be due to poor transduction efficiency (Rainov, 2000).

Toca 511 is a replication-competent retroviral vector that delivers cytosine deaminase (CD) to convert prodrug 5-fluorocytosine (5-FC) to antineoplastic agent 5-fluorouracil (5-FU) (Huang et al., 2013). Toca 511 was tested in 45 high grade glioma patients in a phase I trial that demonstrated a significantly longer OS of 13.6 months compared to control (Cloughesy et al., 2016). These favorable results led to a phase II trial testing Toca 511 to SOC in GBM patients and demonstrated no significant difference in median survival between the two groups (Cloughesy et al., 2020).

### Mechanisms of Resistance

Despite the successes seen with immunotherapy against GBM in preclinical models, these results have not translated well in the clinical setting, as fewer than 10% of GBM patients have been shown to respond to immunotherapy (McGranahan et al., 2019) (p2401). As such, it is important to understand the intrinsic and adaptive forms of resistance exhibited in GBM (Table 5).

**TABLE 5:** Mechanisms of immunotherapy resistance in glioblastoma.

Category of resistance	Example of resistance	References
Primary	CNS immune privilege: 1. BBB 2. CNS lymphatics 3. Resident microglia	(Carson et al., 2006)
	Intrinsic tumor heterogeneity: 1. Classical subtypes 2. Non-classical hybrid cellular states	(Patel et al., 2014)
	Immunosuppressive tumor microenvironment: 1. T cell dysfunction & exhaustion 2. Monocyte/macrophage populations	(Woroniecka et al., 2018) (Marvel and Gabrilovich, 2015)
Secondary	SOC-induced changes: 1. Lymphopenia secondary to TMZ 2. Immunosuppressive cell populations upregulated secondary to dexamethasone	(Gustafson et al., 2010) (Karachi et al., 2018)
	Immunotherapeutic pressure: 1. Expression of alternative checkpoints (TIM3) 2. Epigenetic changes secondary to chronic IFN-γ 3. Loss of tumor antigen expression	(Koyama et al., 2016) (Benci et al., 2016)

### Intrinsic (Primary) Resistance

Several factors contribute to immune evasion in GBM. Unlike other cancers, GBM has been known to display extensive intratumoral heterogeneity (Patel et al., 2014), which confounds efforts to identify high-quality clonal neoantigens regardless of the form of immunotherapy trialed. While GBM has traditionally been classified into proneural, classical, mesenchymal, and neural subsets, single cell RNA-sequencing has revealed cross-over among the subtypes within the same tumor and the presence of hybrid cellular states (Patel et al., 2014). Genomic studies of GBM heterogeneity have revealed the presence of a CD133^+^ chemo- and radio-resistant cancer stem cell (CSC) population responsible for tumor initiation and found to have higher expression in recurrent tumors (Bao et al., 2006; Liu et al., 2006; Tamura et al., 2013; Qazi et al., 2017), but even then, CD133^+^ has failed to be a universal marker of CSCs given the discovery of similarly functioning CD133^-^ cell populations (Chen et al., 2010).

In addition to the extensive phenotypic heterogeneity of cells within a tumor, there is a strong immunosuppressive microenviroment within GBM tumors that remains a major barrier to immunotherapy efficacy (Moserle and Casanovas, 2013). At baseline, GBM patients exhibit a lower number of circulating T cells despite being treatment-naïve (Chongsathidkiet et al., 2018). This phenomenon has been attributed to T cell sequestration in the bone marrow in the setting of GBM as well as other intracranial tumors, due to tumor-mediated internalization of G-protein coupled receptor sphingosine-1-phosphate receptor 1 (S1P1) (Chongsathidkiet et al., 2018). The same study demonstrated reversal of T cell sequestration in murine models of GBM upon inhibiting S1P1 internalization (Chongsathidkiet et al., 2018).

The circulating T cells that are available in the setting of GBM often display dysfunctional phenotypes including but not limited to tolerance and exhaustion (Brooks et al., 1977; Woroniecka et al., 2018). The exhausted phenotype is commonly seen in chronic viral infections and various cancers and has recently been suggested to be irreversible in spite of antigen clearance due to a novel concept referred to as ‘epigenetic scarring’ (Abdel-Hakeem et al., 2021). Persistent proliferation in the setting of chronic antigenic exposure can also lead to shortening of telomeres resulting in T cell senescence (Woroniecka et al., 2018). Importantly, GBM utilizes naturally occurring mechanisms of immune tolerance to promote FasL-mediated peripheral deletion of T cells and recruitment of Tregs via expression of IDO1 on dendritic cells and T cell immunoglobulin- and mucin domain-containing molecule 4 (TIM4) on macrophages (Xu et al., 2011; Choi et al., 2012; Woroniecka et al., 2018). Beyond lymphocytes, the presence of myeloid-derived suppressor cells (MDSC) in both peripheral blood and intracranially has been shown to contribute to immune suppression and tumor progression via expression of arginase, inducible nitric oxide synthase, and reactive oxygen or nitrogen species (Bronte and Zanovello, 2005; Gabrilovich and Nagaraj, 2009; Marvel and Gabrilovich, 2015). Tumor-associated macrophages (TAM) have been similarly implicated via secretion of immunosuppressive cytokines IL10 and TGFβ secondary to induction by CSCs. (Wu et al., 2010; Zhou et al., 2016).

While the central nervous system (CNS) is known to be a site of immune privilege secondary to naturally occurring mechanisms of immune homeostasis such as the blood brain barrier (BBB) and resident microglia (Desland and Hormigo, 2020), the immunosuppression intrinsic to GBM and independent of location in the CNS is clear when comparing the microenvironment and immunotherapeutic results of GBM to that of brain metastases (Friebel et al., 2020). Single cell analysis has revealed that the GBM microenvironment has higher expression of tissue-resident microglia while the metastatic tumor environment has higher expression of tissue-invading leukocytes (Friebel et al., 2020). At the same time, 80% of the leukocytes expressed in the GBM microenvironment were found to be classically immunosuppressive TAMs while most leukocytes found in the metastatic tumor environment were T cells (Friebel et al., 2020). These dichotomous immune findings may help explain why ICIs have seen more success in treating patients with brain metastases from melanoma or NSCLC compared to those with GBM (Goldberg et al., 2016; Tawbi et al., 2018; Kluger et al., 2019).

### Adaptive (Secondary) Resistance

GBM has been shown to acquire forms of secondary resistance in the setting of recurrence and treatment. Patients with rGBM who initially responded to anti-PD1 immunotherapy have been found to have loss of neo-epitopes and delayed upregulation of immunosuppressive genes upon recurrence (Zhao et al., 2019). Despite the expression of EGFRvIII in 19% of ndGBM, a vaccine trial targeting EGFR found that 82% of tumors had lost EGFRvIII expression upon recurrence (Sampson et al., 2010; Brennan et al., 2013).

The current standard of care for GBM can also further exacerbate immune evasion. Dexamethasone, which is commonly used to reduce peri-tumoral edema and temporarily improve neurological symptoms, has been shown to upregulate expression of CTLA-4 on T cells and subsequently dampen patient response to checkpoint blockade (Giles et al., 2018). A unique phenotype of altered monocytes (CD14^+^ HLA-DR^-^) has also been identified to represent an immunosuppressive population whose levels increase in response to dexamethasone treatment (Gustafson et al., 2010). TMZ treatment may also result in profound lymphopenia, and in GBM patients, TMZ-induced lymphopenia is worsened by both the absence of a compensatory increase in proliferation-inducing cytokines and the failure of adoptive transfer to increase T cell counts (Karachi et al., 2018). Radiotherapy alone promotes secretion of classically immunosuppressive cytokines IL-6, IL-8, IL10 (Tabatabaei et al., 2017; Authier et al., 2015) but when combined with chemotherapy as in the current SOC, the combination has been shown to severely deplete CD4^+^ and CD8^+^ T cells and increase the proportion of Tregs (Fadul et al., 2011). In a study of 96 patients with high grade gliomas, patients with CD4^+^ counts under 200 at 2 months after therapeutic initiation were found to have significantly shorter survival than those with higher counts (13.1 vs 19.7 months), highlighting the importance of understanding the effects of treatment in contributing to drug resistance in GBM (Grossman et al., 2011).

The use of immunotherapeutic agents discussed here may also contribute to secondary resistance. In melanoma patients treated with anti-PD1 blockade who subsequently relapsed, genomic analysis of paired primary and recurrent tumor revealed alterations in β2 microglobulin and JAK1/2 genes that were the main drivers of acquired PD1 resistance (Shin et al., 2017). It has also been shown in lung cancer that downregulation of one checkpoint may lead to subsequent upregulation of other checkpoint molecules, such as the upregulation of TIM3 in the setting of anti-PD1 blockade (Koyama et al., 2016). In glioblastoma, the use of CAR T cells targeting EGFRvIII has been linked to a compensatory influx of immunosuppressive Tregs into the tumor microenvironment as well as loss of EGFRvIII expression in surgically resected tumors post-treatment (O’Rourke et al., 2017). Similarly, the use of CAR T cells targeting IL13Rα2 was shown to lead to improved survival in preclinical murine models, but recurrent gliomas post-treatment experienced downregulation of the target antigen, suggesting that the use of CAR T cells targeting a singular antigen may select for GBM cells that lack expression and subsequently allow for disease progression (Krenciute et al., 2017).

## Overcoming Resistance Mechanisms to Immunotherapy (Table 6)

The limited clinical success seen in the use of immunotherapeutic agents against GBM is likely due to a multifactorial process of immunosuppression, local immune cell dysfunction, and tumor cell heterogeneity, as highlighted by the aforementioned mechanisms of tumor resistance. As a result, adjuvant approaches that prime the tumor microenvironment for a robust, antitumor immune response have been the focus of active investigation (Lim et al., 2018). In this final section, we will highlight strategies that remodel the tumor microenvironment for immunotherapy by downregulating its immunosuppressive qualities and upregulating its cytolytic potential, as well as their potential and/or observed adverse effects.

**TABLE 6:** Immunotherapy sensitization strategies in glioblastoma.

Target molecule	Target cell	Combinatorial treatments for maximal effect	Expected blockade effect	Potential adverse events	Clinical trial	Phase
CD47	GBM	Anti-PD1 (von Roemeling et al., 2020)	Reduce tumor burden by stimulating M1 macrophage-mediated phagocytosis	Hematological Toxicity (Jalil et al., 2020)	NA	NA
CSF-1R	Macrophage	Anti-PD1 (Antonios et al., 2017)	Functionally reprogram macrophages from M2 to M1 polarization	Hepatotoxicity (Butowski et al., 2014; Papadopoulos et al., 2017; von Tresckow et al., 2015)	NCT02526017 (Brahmer et al., 2016)	I
CD73	Macrophage	Anti-PD1 and Anti-CTLA-4 (Goswami et al., 2020)	Inhibit production of tumorigenic adenosine from M2 macrophages	NA	NA	NA
PGE2	MDSC	Anti-PD1 (Hou et al., 2016)	Inhibit the expansion of MDSCs	Autoimmunity (Yang et al., 2017)	NA	NA
CCL2	MDSC	Anti-PD1 (Flores-Toro et al., 2020)	Reduce the recruitment of MDSCs into the tumor microenvironment	Neutropenia (Brana et al., 2015; Gschwandtner et al., 2019)	NA	NA
MIF	MDSC	Anti-PD1 (Alban et al., 2020)	Inhibit the induction of MDSCs in the tumor microenvironment	Gastrointestinal Distress (Rolan et al., 2008; Fox et al., 2018)	NCT03782415 (MediciNova, 2021)	I/II
IL-6	MDSC	CD40 Stimulation Anti-PD1 Anti-CTLA4 (Yang et al., 2021)	Reduce the recruitment of MDSCs and prevent polarization of myeloid cells towards M2 phenotype	Neutropenia and thrombocytopenia (Wright et al., 2014)^(p6),^ (Smolen et al., 2013)	NCT04729959 (National Cancer Institute NCI, 2021)	II
GITR	Tregs	Anti-PD1 (Wu et al., 2019) (p)	Reprogram Tregs into CD4^+^ T cells	Autoimmunity (Sakaguchi et al., 2006)	NCT04225039 (University of Pennsylvania, 2021)	II

Abbreviations; OS, overall survival; PFS, Progression-free survival; NR, not reported; RT, radiotherapy; TMZ, temozolomide; ndGBM, newly diagnosed GBM; rGBM, recurrent GBM; NA, not applicable.

### Anti-CD47

Many efforts have been directed towards targeting the myeloid compartment, specifically M2-type TAMs and MDSCs, given their wide variety of immunosuppressive functions and dense abundance in the tumor mass (Glass and Synowitz, 2014). CD47, a ubiquitously expressed protein on the surface of GBM cells, interacts with signal-regulatory protein alpha (SIRP α ) on the membranes of macrophages to inhibit phagocytosis by promoting M2 polarization through the PI3K/AKT signaling pathway (Lin et al., 2018; Liu et al., 2020). Inhibiting the CD47/SIRPα anti-phagocytic and pro-M2 axis in GBM has shown promising results by shifting macrophages towards the antitumorigenic M1 phenotype, reducing tumor burden by enhancing macrophage-mediated phagocytosis, and improving survival in xenograft mouse models (Zhang et al., 2016; Gholamin et al., 2017). While anti-CD47 monotherapy has since been shown to be inefficient in immune competent hosts, combinatorial treatment of CD47 blockade and TMZ has led to augmented PD-1 blockade responses and improved survival in murine models (von Roemeling et al., 2020). Its potential and preliminary success as a target for combination anti-PD-1 immunotherapy is hypothesized to be due to increased phagocytosis of tumor cells and induction of the endoplasmic stress response that results in more efficient T cell priming (von Roemeling et al., 2020).

CD47 inhibitors have been tested in several phase I/II clinical trials for hematological and advanced solid malignancies (Jalil et al., 2020). While preclinical studies in mice have shown good tolerance with minimal signs of toxicity, clinical trials have encountered issues regarding hematological toxicity including anemia, leukopenia, and thrombocytopenia (Gholamin et al., 2017; Li et al., 2018). CD47 is ubiquitously expressed by non-cancerous cells of the hematopoietic system, which makes them an alternative binding site for systemically delivered anti-CD47 antibodies (Ishikawa-Sekigami et al., 2006; Hu et al., 2020). Consequently, non-tumor binding of antibodies was found to induce unintentional FcR-mediated phagocytosis of red blood cells leading to toxic anemia (Zhang et al., 2020). Studies have experienced success in mitigating hematological toxicity by delivering a low, priming dose of anti-CD47 antibodies to induce a predictable, transient anemia and compensatory reticulocytosis for non-Hodgkin’s lymphoma (Advani et al., 2018). However, future investigations of applying anti-CD47 treatment to GBM must still consider the possibility of hematological toxicity and develop effective strategies to bypass it.

### Anti-CSF-1R

Similar to CD47, colony stimulating factor-1 receptor (CSF-1R), a member of the receptor protein tyrosine kinase (rPTK) family, has also been implicated in the differentiation of myeloid cells into M2 macrophages (Dai et al., 2002). In GBM xenograft models, CSF-1R inhibition decreases M2 markers on TAMs but does not deplete the cell population due to tumor-secreted factors, notably GM-CSF and IFN- γ (Pyonteck et al., 2013). However, blockade of CSF-1R has also been shown to increase expression of key chemotactic factors that promote the influx of tumor-infiltrating lymphocytes, a finding consistent with other cancer types (Mok et al., 2014; Ries et al., 2014; Antonios et al., 2017). When inhibition was performed in conjunction with anti-PD-1 and DC vaccination, TIL dysfunction was reversed, suggesting that the development of immune resistance to active vaccination in GBM can be abrogated by combinatorial inhibition of CSF-1R and PD-1 (Antonios et al., 2017). Clinical trials with cabiralizumab, an anti-CSF-1R monoclonal antibody, are currently underway for malignant glioma in conjunction with the anti-PD-1 antibody nivolumab (Brahmer et al., 2016). They have also been performed for other cancers, with a recent phase 1 study in anti-PD-L1 resistant patients with melanoma, kidney cancer, or non-small lung cancer exhibiting low clinical response rates but safe upregulation of pro-inflammatory cytokines and chemokines (Weiss et al., 2021). Overall, CSF-1R inhibitors have been generally well-tolerated, increasing its promise for combinatorial immunotherapy strategies (Cannarile et al., 2017). However, short-lived, asymptomatic elevations in liver enzymes, notably alanine transaminase, aspartate aminotransferase, creatine kinase, and lactate dehydrogenase, have been observed across multiple different studies for different cancers, including GBM (Butowski et al., 2014; Papadopoulos et al., 2017; von Tresckow et al., 2015). Hepatotoxicity is believed to be due to partial depletion of CSF-1R + macrophages/Kupffer cells of the liver leading to reduced physiologic clearance (Ries et al., 2014). While no functional or structural liver damage has been associated with anti-CSF-1R treatment, careful monitoring will be required in future studies.

### Anti-CD73

In addition to targeting the polarization of M2 macrophages, potential adjuvant treatments have also aimed to target the function of M2-type macrophages. Specifically, CD73, an ectonucleotidase preferentially expressed on M2 polarized macrophages, interacts with its upstream signaling molecule CD39 to facilitate the production of adenosine from extracellular ATP (Zanin et al., 2012; Antonioli et al., 2013). Adenosine is a known promoter of tumor proliferation and angiogenesis, and in the case of GBM, it has been implicated in the development of TMZ chemoresistance and CD8^+^ T cell dysfunction via the A_2B_ adenosine receptor (Takenaka et al., 2019; Yan et al., 2019). CD73 expression on myeloid cells has also been correlated with higher co-expression of the immunosuppressive and protumorigenic CCR2, CCR5, ITGAV, and CSF-1R chemokine receptors (Goswami et al., 2020). Clinical trials targeting these chemokine receptors are underway, but CD73 may be a more relevant target given its high co-expression and the limited success observed thus far. A preliminary study that induced prolonged survival in CD73 knockout models of GBM treated with dual blockade of PD-1 and CTLA-4 highlights the potential of CD73 for combination therapy and need for further investigation (Goswami et al., 2020). Given that clinical trials focusing on targeting CD73 in GBM have yet to commence or are ongoing in other malignances, there is limited awareness of its associated adverse events beyond results from animal studies (Jin et al., 2021). While studies in mice have shown that CD73 plays a role in platelet aggregation and protection of the heart, kidney, and lungs from ischemia, animal studies have shown good tolerability to CD73 blockade (Stagg, 2012; Antonioli et al., 2016; Azambuja et al., 2020). However, these results should not be used to extrapolate responses in humans, and a better understanding of the safety of targeting CD73 will be had following the conclusion of ongoing clinical trials.

### Cyclooxygenase-2 (COX-2) Inhibitors

Myeloid-derived suppressor cells (MDSC), a heterogenous population of CD11b^+^ CD33^+^ HLA-DR^-^ myeloid cells found in the peripheral blood and tumor mass of GBM patients, have also emerged as a potential target for sensitizing the tumor microenvironment to immunotherapy given their myriad immunosuppressive functions associated with poor prognosis (Almand et al., 2001; Alban et al., 2018). MDSCs have been implicated in GBM tumor progression through the inhibition of T cells, NK cells, dendritic cells, and macrophages, the expansion and differentiation of T regulatory cells, and the promotion of immunosuppressive B cells (Raychaudhuri et al., 2011; Mi et al., 2020). To control their tumorigenic activity, approaches have attempted to target the infiltration, expansion, and activation of MDSCs. Prostaglandin E2 (PGE2) and C-C motif chemokine ligand 2 (CCL2) have both been associated with the recruitment of MDSCs to tumor tissue, while macrophage migration inhibitory factor (MIF) signaling through the chemokine ligand 2 (CXCL2) and MIF/C-X-C motif chemokine receptor 2 (CXCR2) axis has been linked to the differentiation of myeloid cells into MDSCs (Simpson et al., 2012; Chang et al., 2016).

In preclinical models, inhibition of PGE2 production through COX2 inhibitors, specifically acetylsalicylic acid and celecoxib, led to both the suppression of gliomagenesis and reduction of MDSCs in the tumor microenvironment through a decrease in CCL2 (Fujita et al., 2011; Shono et al., 2020). Furthermore, COX2 inhibition in GBM has also garnered interest for its radiosensitizing effects *in vivo* (Ma et al., 2011). While the enthusiasm for COX2 as a therapeutic target has stagnated due to multiple cohort studies and clinical trials that inversely correlated COX2 inhibitors with survival, its efficacy as an immunotherapeutic adjuvant has yet to be thoroughly investigated and warrants further study (Qiu et al., 2017). Alternatively, it may also be appropriate to shift focus towards PGE2 as a therapeutic target rather than COX2 itself, especially given the broad range of well-known adverse effects associated with COX2 inhibition such as hypertension, congestive heart failure exacerbation, renal impairment, and other cardiovascular events (Mukherjee et al., 2001; Wright, 2002). There are currently no clinical trials focused on targeting PGE2. However, an oncolytic vaccina virus was recently designed to inactivate PGE2 and successfully reduce the number of MDSCs and T regulatory cells in a mouse tumor model as well as potentiate the response to anti-PD1 therapy (Hou et al., 2016). Thus, the safety associated with targeting PGE2 is currently unknown beyond those associated with COX2 inhibition, however, downregulating MDSCs will pose the risk of autoimmune side-effects such as autoreactive T-cells, more so than traditional immunotherapies given the wider spectrum of activity in MDSCs than targeted immune checkpoint inhibitors (Yang et al., 2017).

### Anti-CCL2

CCL2 inhibition through anti-CCL2 monoclonal antibodies (mAb) has also experienced success in reducing the population of MDSCs and improving survival in GBM xenograft models (Zhu et al., 2011). Additionally, inhibition of the CCL2 receptor (CCR2) in conjunction with PD-1 checkpoint inhibition extended survival in GBM-bearing mice, highlighting its ability to augment immunotherapy (Flores-Toro et al., 2020). Clinical trial using carlumab monotherapy, a human IgG1_k_ monoclonal antibody against CCL2, had little success in patients with advanced solid tumors and metastatic prostate cancer, however, results from CCL2 blockade with immune checkpoint blockade have yet to be reported (Pienta et al., 2013; Sandhu et al., 2013). While carlumab clinical trials were well-tolerated with mild-to-moderate adverse events, neutropenia occurred commonly in a multicenter phase 1b study, which may be due to the elimination of CCL2’s anti-apoptotic effect in neutrophils and increase the risk of infection in patients (Brana et al., 2015; Gschwandtner et al., 2019).

The MIF signaling axis has also recently garnered interest as a target given the high levels of the MIF non-cognate receptor CXCR2 expressed on MDSCs and the enhanced CD8^+^ T cell activity observed in the tumor microenvironment following treatment with ibudilast, an inhibitor of MIF-CD74 interactions (Alban et al., 2020). Ibudilast is currently being investigated in clinical trials as an adjuvant to TMZ, given its ability to sensitize GBM cells to TMZ, safely penetrate the BBB, and confer minimal adverse events, with phase 1 studies and clinical trials for other neurological diseases such as multiple sclerosis reporting gastrointestinal side effects, headaches, and depression as the most concerning (Rolan et al., 2008; Fox et al., 2018). However, given the preliminary findings of increased lymphocyte activation, additional clinical trials pairing ibudilast with immune checkpoint inhibitors like anti-PD-1 to maximize cytolytic activity against tumor cells should also be considered.

### Anti-IL-6

IL-6 drives myeloid-based immunosuppressive activity through the induction of PD-L1 expression on MDSCs (Lamano et al., 2019). While IL-6 neutralization has been shown to enhance T cell tumor infiltration, it alone does not sensitize the tumor to immune checkpoint blockade via anti-PD-1 or anti-CTLA-4 (Yang et al., 2021). However, CD40 stimulation in conjunction with IL-6 inhibition and PD-1 and CTLA-4 immune checkpoint blockade did produce clinically relevant responses in syngeneic GBM models, notably the reversal of macrophage-mediated immune suppression and extended survival Yang et al., 2021. This finding supporting dual-targeting of IL-6, CD40, and multiple immune checkpoints emphasizes the paradigm shift towards strategically selecting multiple interrelated targets that increase the possibility of a successful response to immunotherapy. An ongoing clinical trial investigating the addition of tocilizumab, a monoclonal antibody against IL-6, alongside atezolizumab and fractionated stereotactic radiation therapy in recurrent GBM will shed more light on the clinical efficacy of concurrent IL-6 and PD-L1 blockade in GBM. Given the use of tocilizumab for other conditions such as rheumatoid arthritis, adverse effects are minimal, acceptable, and promising for utilization in GBM treatment. Complications mainly consist of neutropenia and rare thrombocytopenia given IL-6 receptors on neutrophils that may bind monoclonal antibodies and lead to opsonization and neutrophil phagocytosis (Wright et al., 2014) (p6), (Smolen et al., 2013).

### Anti-Glucocorticoid-Induced TNFR-Related Protein

In addition to cells from the myeloid compartments, cells derived from the lymphoid lineage also possess immunosuppressive functions and serve as potential therapeutic targets, most notable of which are Tregs. Tregs are characterized as a CD25^+^ FOXP3^+^ subset of CD4^+^ cells that divert immune responses away from cytotoxic Th1-mediated responses and towards Th2-mediated responses in part through increasing the expression of CTLA-4 and decreasing the secretion of IL-2 and IFN- γ (Jonuleit et al., 2001; Cosmi et al., 2004). To improve the antitumor immune response, Treg depletion therapy has been attempted in GBM models through combinatorial anti-CXCR4 and anti-PD1 immunotherapy, which have improved survival rates via decreased levels of Tregs and MDSCs, improved CD4^+^/CD8^+^ ratios, and increased levels of pro-inflammatory cytokines (Wu et al., 2019). However, systemic Treg depletion through antibody therapies pose the risk of autoimmunity (Sakaguchi et al., 2006). Thus, recent findings targeting GITR, a surface immunomodulatory receptor highly expressed on GBM Tregs but lowly expressed on systemic Tregs, are especially encouraging for Treg depletion therapies. When targeting GITR, murine Tregs were reprogrammed into CD4^+^ Th1 cells capable of producing IFN- γ and engaging in cytotoxic activity against GBM cells (Amoozgar et al., 2021). Furthermore, anti-GITR and anti-PD1 combinatorial treatment was capable of prolonging survival in multiple GBM murine models, with a subset experiencing complete tumor eradication and immune memory following tumor re-challenge (Miska et al., 2016; Amoozgar et al., 2021). Dual radiation and anti-GITR therapy has also been associated with improved survival via increased CD4^+^ effector T cell infiltration and IFN- γ , IL-2, and TNF- α secretion, suggesting that the two modalities synergize and highlighting the immune sensitizing effects of radiotherapy on the tumor microenvironment (Patel et al., 2016). A clinical trial investigating the use of anti-GITR, anti-PD1, and stereotactic radiosurgery in recurrent GBM was recently initiated and will provide valuable insight into how and to what extent the tumor immune microenvironment responds to treatment.

### Other Forms of Sensitization

Despite its immunosuppressive mechanisms documented earlier, radiotherapy can also sensitize the immune response via its ability to increase MHC-1 expression on the surface of tumor cells, leading to better antigen presentation and recognition by cytotoxic T cells (Rajani et al., 2019; Sevenich, 2019). Antiangiogenic therapies have also been considered as a treatment option for overcoming resistance to immune checkpoint therapies in GBM. Dual blockade of VEGF and Ang-2 with concurrent anti-PD-1 treatment has been shown to extend survival, increase cytotoxic T lymphocyte infiltration, and decrease MDSC and Treg abundance (Fukumura et al., 2018; Di Tacchio et al., 2019).

Overall, clinical trials investigating multimodal therapies in GBM that integrate conventional treatments, such as radiotherapy and anti-angiogenic therapy, with novel immunotherapies and strategies for bypassing tumor resistance are currently underway. This combinatorial approach to treatment exemplifies the new frontier of GBM immunotherapy that leverages strategic combinations of multiple treatments to reverse immunosuppression within the microenvironment and maximize the potential of immunotherapy.

## Conclusion

Much progress has been made in the landscape of immunotherapies designed to treat glioblastoma. Several immune checkpoint inhibitors have been adopted from other cancer trials for use in glioblastoma, and although the uniquely resistant environment of glioblastoma has prevented similar successes seen in other cancers, several advances have been made in introducing new vaccine, adoptive T cell, and oncolytic virotherapies to induce both tumor lysis and an anti-tumor immune response. New forms of sensitization to overcome primary and adaptive resistance include myeloid and lymphoid-targeting strategies, as well as the introduction of multimodal treatments integrated with conventional standard of care. Looking forward, it is important to recognize the intrinsic differences between glioblastoma and other cancers that the aforementioned therapies have been trialed in, in order to design more targeted treatments that can overcome the uniquely immunosuppressive environment of GBM.
